# The story vs the storyteller: Factors associated with the effectiveness of brief video‐recorded patient stories for promoting opioid tapering

**DOI:** 10.1111/hex.13243

**Published:** 2021-04-09

**Authors:** Stephen G. Henry, Bo Feng, Susan Verba, Richard L. Kravitz, Ana‐Maria Iosif

**Affiliations:** ^1^ Department of Internal Medicine and Center for Healthcare Policy and Research University of California, Davis Sacramento CA USA; ^2^ Department of Communication University of California, Davis Davis CA USA; ^3^ Department of Design and Center for Design in the Public Interest University of California, Davis Davis CA USA; ^4^ Department of Public Health Sciences University of California, Davis Davis CA USA

**Keywords:** chronic pain, narrative transportation theory, opioid analgesics, patient education, persuasive communication, tapering

## Abstract

**Background:**

Narrative communication is often more persuasive for promoting health behaviour change than communication using facts and figures; the extent to which narrative persuasiveness is due to patients’ identification with the storyteller vs engagement with the story is unclear.

**Objective:**

To examine the relative impacts of patient engagement, age concordance and gender concordance on perceived persuasiveness of video‐recorded narrative clips about opioid tapering.

**Methods:**

Patient raters watched and rated 48 brief video‐recorded clips featuring 1 of 7 different storytellers describing their experiences with opioid tapering. The dependent variable was clips’ perceived persuasiveness for encouraging patients to consider opioid tapering. Independent variables were rater engagement with the clip, rater‐storyteller gender concordance and rater‐storyteller age concordance (<60 vs ≥60). Covariates were rater beliefs about opioids and opioid tapering, clip duration and clip theme. Mixed‐effects models accounted for raters viewing multiple clips and clips nested within storytellers.

**Results:**

In multivariable models, higher rater engagement with the clip was associated with higher perceived persuasiveness (coefficient = 0.46, 95% CI 0.39‐0.53, *P* < .001). Neither age concordance nor gender concordance significantly predicted perceived persuasiveness. The theme *Problems with opioids* also predicted perceived persuasiveness.

**Conclusion:**

Highly engaging, clinically relevant stories are likely persuasive to patients regardless of the match between patient and storyteller age and gender. When using patient stories in tools to promote health behaviour change, stories that are clinically relevant and engaging are likely to be persuasive regardless of storytellers’ demographics.

**Patient or public contribution:**

Patients were involved as storytellers (in each clip) and assessed the key study variables.

## 1 INTRODUCTION

Narrative, or story‐based, communication is often more effective for promoting health behaviour change than didactic communication using facts and figures. For example, randomized clinical trials comparing narrative vs non‐narrative patient education videos have found that narrative videos resulted in significantly better blood pressure control among African American patients with uncontrolled hypertension.^1^ A randomized trial comparing narrative vs non‐narrative films promoting cervical cancer screening found that women in both arms demonstrated improvements in knowledge and attitudes about cervical cancer screening. These improvements were significantly greater for women who watched the narrative video (vs the non‐narrative video), and only women who watched the narrative video were significantly more likely to have undergone or scheduled cervical cancer screening 6 months later.^2^ Narrative transportation theory posits that the persuasiveness of narrative communication is driven by the extent to which a narrative's audience feel ‘transported’ into the story while processing it, and that greater transportation is driven by emotional engagement with the narrative, identification with the storyteller and perceptions of narrative authenticity.^3^, ^4^, ^5^ Recent meta‐analyses have concluded that narrative messages decrease resistance significantly more than non‐narrative ones^6^ and that narratives delivered via video or audio are typically more persuasive than written narratives.^7^ However, literature on the effects of similarity between storytellers and patients on persuasiveness, including the effect of gender and age concordance, is mixed.^8^ Ooms et al examined the effects of gender and age concordance on participants’ intent to conduct cancer self‐examinations after reading narrative health messages about gender‐related cancers (ie breast or testicular cancer). They found that younger student participants identified significantly more with younger (vs older) storytellers, but that neither age nor gender concordance was associated with intent to perform cancer‐related self‐examinations or donate to cancer charities.^9^ In contrast, when Chen et al conducted a similar experimental study to examine factors associated with perceived persuasiveness of a story about diabetes, gender concordance and age concordance were strongly associated with both participants’ identification with the storyteller and the story's perceived persuasiveness.^10^

Understanding the impact of age and gender concordance on narrative persuasiveness is particularly important in health communication, where there is a need for brief, effective interventions that can be incorporated into clinical workflows to promote patient health behaviour change. Patient education videos are commonly used in clinical studies, and a systematic review of such videos found that videos delivering story‐based messages were more persuasive than non‐narrative videos.^11^ Storytellers’ age and gender are easy to assess and so are often manipulated by health researchers as a way to increase patients’ identification with a story and, by extension, its perceived persuasiveness.^1^, ^12^ Further research on the extent to which age concordance and gender concordance affect the perceived persuasiveness of narrative videos could inform future studies and health intervention development by clarifying the extent to which matching patient and storyteller demographics is an effective strategy for increasing persuasiveness. We also know little about the extent to which persuasiveness is associated with patients’ identification with the storyteller vs their engagement with the story overall.

To examine the relative contribution of these factors, we analysed patient ratings of brief video clips showing patient stories that were collected to create a patient education video about opioid tapering. We focused on opioid tapering because reducing rates of opioid‐related harms (including overdose and opioid use disorder) is an urgent public health priority in the United States.^13^ In addition, reviews have found that narrative messages tend to be less effective at changing intention when encouraging behavioural cessation (eg reduce opioid consumption) vs when encouraging patients to initiate preventive or health screening behaviours.^7^ Thus, identifying factors associated with perceived persuasiveness is particularly important for behavioural cessation messages, because researchers need as many tools as possible to optimize the persuasiveness of behavioural cessation messages when designing videos or other health interventions. Our primary objective was to investigate whether patients’ engagement with the video clip, gender concordance between patient and storyteller, and age concordance between patient and storyteller were associated with patients’ ratings of stories’ overall persuasiveness. We also examined other factors that could impact stories’ persuasiveness, including patients’ attitudes towards opioids and opioid tapering, clip duration and clip theme (ie narrative content).

## 2 METHODS

### 2.1 Study design and population

This study was part of a larger project to create a patient education video about opioid tapering. We recruited both compelling storytellers (who were featured in narrative video clips) and raters who evaluated those clips. Storytellers and raters were recruited in a 2‐phase process from the same population using identical recruitment procedures and eligibility criteria. All participants were adults at 13 primary care clinics in Northern California who reported moderate‐to‐severe chronic neck and/or back pain and had either tapered down or off long‐term prescription opioids (defined as at least one opioid dose per day for at least three months) within the past year. We recruited patients aged 35‐85 years because our prior research found very few patients younger than 35 who took long‐term opioids for chronic back or neck pain.^14^ Participant exclusion criteria were pregnancy, active cancer treatment, being enrolled in hospice or palliative care, and being prescribed opioids by specialists rather than primary care clinicians. We used an electronic health record screening algorithm to identify potentially eligible patients who met these criteria, and then gave primary care clinicians lists of their patients identified by the algorithm and asked clinicians to identify their patients who were good candidates for opioid tapering, were in the process of tapering or had finished tapering within the past 12 months. Study personnel independently contacted the identified patients, assessed their eligibility via telephone and then invited interested patients to participate.

#### 2.1.1 Storyteller identification and clip selection

To identify compelling storytellers, in the first phase of recruitment we enrolled 21 participants who took part in 4 focus groups about opioid tapering (eighty‐nine percent of patients who were screened and eligible for inclusion agreed to participate).^15^ During each focus group, an investigator not conducting the focus group acted as an observer and took notes on group dynamics. Researchers then identified compelling storytellers by reviewing focus group audio recordings and transcripts, and notes from focus group observers. Compelling storytellers were defined as participants who told stories about their experiences with opioid tapering that seemed authentic, coherent and engaged or kept the attention of other focus group participants. We invited 9 compelling storytellers to participate in an additional 30‐minute 1‐on‐1 video‐recorded interview, during which time they would recount and elaborate on their personal experiences with opioid tapering. Two participants declined to be interviewed (1 due to concerns about being video‐recorded and 1 due to scheduling conflicts), leaving seven compelling storytellers. From these interviews, investigators selected 48 brief video clips (mean duration 42 seconds; SD = 12; range 19‐70 seconds) for possible inclusion in the patient education video.

To select clips, researchers viewed all interviews and identified video segments that showed a storyteller recounting a brief, coherent story about their experiences related to opioid tapering. Consistent with the overall project goal, we did not select clips that explicitly discouraged opioid tapering or explicitly encouraged opioid dose escalation. All identified segments were edited into clips that were stored as separate digital files. Two examples of video clips rated in this study can be viewed in the Videos S1 and S2.

#### 2.1.2 Clip ratings

For the second phase, patient raters were recruited from the same clinics using the same methods and eligibility criteria as storytellers. We began recruiting raters when we had almost finished conducting focus groups. Participants who were unable to participate in focus groups were eligible to participate as raters. However, focus group participants were not eligible to be raters. In addition, rater recruitment was stratified by age (<60 vs ≥60 years) and gender. To our knowledge, no raters were acquainted with any of the storytellers.

Each rater rated 24 randomly selected narrative clips, for a total of 1152 ratings (12 ratings per clip; 3 per gender‐by‐age category). The 48 clips were first randomly divided into two groups (‘A’ and ‘B’) of 24 clips each. Raters were then scheduled to watch the video clips in a series of small groups. Each small group watched either the 24 clips in group ‘A’ or the 24 clips in group ‘B’. During each small group, raters were first informed of the study purpose and then watched and rated the 24 video clips on several Likert‐type items. Raters watched each clip and then immediately recorded their ratings for that clip using paper questionnaires. Rating all 24 clips took approximately 90 minutes; raters were given a break midway through to prevent fatigue. Raters were seated to ensure that they could not observe how others rated the clips. During each small group, raters viewed clips in a different random order (using a random order generated by the study biostatistician) to minimize potential order effects. Raters were assigned to small groups to ensure balanced recruitment for each gender‐by‐age category. Prior to viewing the clips, raters provided data on demographic characteristics (Table 1) and attitude towards opioids (Table 2).

**TABLE 1:** Rater characteristics (n = 48)

Age (y), mean (SD)	58.8 (9.0)
Male gender, n (%)	24 (50%)
Race, n (%)	
African American	1 (2%)
Caucasian	43 (90%)
Other^a^	4 (8%)
Hispanic, n (%)	7 (15%)
Status of opioid tapering, n (%)	
Finished tapering within past year	18 (38%)
In the process of tapering	14 (29%)
Clinician had recommended but not yet started tapering	8 (17%)
Clinician had not recommended tapering	8 (17%)
Highest education attained, n (%)	
High school or less	5 (10%)
Some college	16 (33%)
AA/technical degree	9 (19%)
Bachelor's degree	13 (27%)
Master's, doctoral or professional degree	5 (10%)
Employment, n (%)	
Self‐employed	3 (6%)
Full time	13 (27%)
Part time	1 (2%)
Out of work	2 (4%)
Not able to work	8 (17%)
Retired	18 (38%)
Other	3 (6%)
Annual household income, n (%)	
<$40 000	10 (21%)
$40k‐$60 000	12 (25%)
$60k‐$80 000	6 (13%)
$80 000‐$100 000	10 (21%)
>$100 000	10 (21%)
Average pain severity,^b^ mean (SD)	6.5 (1.9)
How long you have been suffering from chronic pain?^c^ n (%)	
<6 mo	2 (4%)
2 y‐5 y	3 (6%)
5 y‐10 y	12 (26%)
≥10 y	30 (64%)

Note

Due to rounding, percentages might not sum to 100.

^a^ Includes biracial, Mexican American, Greek and Indian.

^b^ Measured using the PEG scale (range 0‐10, with higher numbers reflecting more severe pain during the past week).^26^

^c^ Data missing for 1 rater.

**TABLE 2:** Rater beliefs about opioids (n = 48)

**Attitudes about opioid tapering**	
I have wanted to stop using opioid pain medicines or to cut down on the amount of opioid medicines that I use,^a^, ^b^ n (%)	
Strongly disagree	3 (6%)
Disagree	7 (15%)
Neutral	14 (30%)
Agree	10 (21%)
Strongly agree	9 (19%)
N/A (not taking opioids)	4 (9%)
**Beliefs about opioid effectiveness**	
How effective are opioid medications at reducing your level of pain?^a^ n (%)	
Not at all	2 (4%)
A little	12 (26%)
Moderately	10 (21%)
Very	13 (28%)
Extremely	6 (13%)
N/A (not taking opioids)	4 (9%)
How effective are opioid medications at reducing how much pain interferes with your normal work or other activities?^a^ n (%)	
Not at all	3 (6%)
A little	8 (17%)
Moderately	20 (43%)
Very	6 (13%)
Extremely	6 (13%)
N/A (not taking opioids)	4 (9%)
**Beliefs about opioid side‐effects**	
During the past 30 d, how much of your time was spent thinking about opioid medications?^c^ n (%)	
Never	7 (15%)
Seldom	11 (23%)
Sometimes	10 (21%)
Often	11 (23%)
Very often	5 (10%)
N/A (not taking opioids)	4 (8%)
During the past 30 d, how often have you been worried about how you are handling your medication?^c^ n (%)	
Never	15 (31%)
Seldom	15 (31%)
Sometimes	8 (17%)
Often	4 (8%)
Very often	2 (4%)
N/A (not taking opioids)	4 (8%)
During the past 30 d, how often have others been worried about how you are handling your medications?^c^ n (%)	
Never	26 (54%)
Seldom	10 (21%)
Sometimes	4 (8%)
Often	4 (8%)
Very often	0 (0%)
N/A (not taking opioids)	4 (8%)
Considering the side‐effects of opioid medicines you experienced in the past month, how bothersome were these side‐effects^a^, ^b^ n (%)	
Not at all	21 (45%)
A little	13 (28%)
Moderately	7 (15%)
Very	2 (4%)
Extremely	0 (0%)
N/A (not taking opioids)	4 (8%)

Note

Due to rounding, percentages might not sum to 100.

^a^ Data missing for 1 rater.

^b^ Item taken from the Prescribed Opioid Difficulties Scale.^16^

^c^ Item taken from the Current Opioid Misuse Measure.^17^

### 2.2 Measures

Raters’ baseline questionnaires included the following *rater characteristics*: age, gender, race, ethnicity (Hispanic vs non‐Hispanic), highest education attained, employment status, annual household income (US dollars), average pain severity, duration of chronic pain and opioid tapering status (finished tapering within the past year, in the process of tapering, clinician had recommended but not yet started tapering, or clinician had not recommended tapering).

Baseline questionnaires also included *covariates* related to raters’ beliefs about opioids and opioid tapering (which could affect perceived persuasiveness). Raters’ attitudes about opioid tapering were measured using 1 item from the Prescribed Opioid Difficulties Scale^16^ (Table 2) analysed as a binary variable (disagree or strongly disagree with desire to taper (reference) vs agree, strongly agree or already tapered). Beliefs about opioid effectiveness were measured by the mean of 2 items asking about opioid effectiveness (Table 2, Cronbach's alpha = 0.95). Beliefs about opioid‐related side‐effects were measured by the mean of 4 items from the Prescribed Opioid Difficulties Scale and the Current Opioid Misuse Measure^17^ (Table 2, Cronbach's alpha = 0.90).

Our *dependent variable* was perceived clip persuasiveness, which raters assessed after viewing each clip by answering the question, ‘After watching this clip, how willing do you think other people with chronic pain would be to try taking less opioid pain medication?’ (rated from 1 = ‘not at all willing’ to 5 = ‘very willing’; mean rating 2.8; SD 0.3). We designed this variable using this particular wording because the planned patient education video was intended to target patients who were candidates for tapering, and we thought that asking patients in varying stages of the tapering process about the clips’ perceived persuasiveness for others would produce more reliable and relevant information.

Our *independent variables* were patient engagement with the clip, age concordance between rater and storyteller, and gender concordance between rater and storyteller. Raters assessed their engagement with each clip after viewing it by answering 5 items related to engagement (rated from 1 = ‘not at all’ to 5 = ‘very’; see Table 3) that we adapted from items used by other research teams to assess the persuasiveness of narrative videos. Engagement was operationalized as the mean of these 5 items (Cronbach's alpha = 0.87). Age concordance and gender concordance were operationalized as binary variables indicating whether the rater and storyteller were in the same age category (<60 vs ≥60 years) and gender category, respectively.

**TABLE 3:** Items used for assessing rater engagement with video clips^a^

Item	Mean (SD)
How engaging was this clip?	3.7 (0.3)
How relevant were the events in this clip to your everyday life?	3.0 (0.5)
How genuine is the person in this clip?	3.7 (0.4)
How much did you like the person in this clip?	3.5 (0.5)
I could understand why the person felt the way he/she felt.	4.0 (0.3)

^a^ Items were rated on a 5‐point scale, with higher scores indicating greater engagement. For example, scores for the first item were 1 = ‘not engaging’, 2 = ‘a little engaging’, 3 = ‘neutral’, 4 = ‘engaging’ and 5 = ‘very engaging’. Reported summaries are calculated after first averaging all 24 ratings for each clip.

Finally, covariates for *clip characteristics* were clip duration (in seconds) and clip theme. To evaluate clip theme, two investigators first viewed each clip and then coded whether the story in that clip included any of the following nine topics: reasons for tapering, opioid‐related risks, fears about tapering, benefits of tapering, communicating with clinicians, managing pain, managing opioids, getting through the day (ie completing daily activities while tapering) and support for tapering (ie support from family or friends). Disagreements were resolved by discussion. Topics were not mutually exclusive; the story in a single clip could be coded for more than one topic (median = 3, range 1 to 5). The most common topics were managing pain (52%) and getting through the day (52%), followed by reasons for tapering (40%). The least common topics were fears about tapering (8%) and benefits of tapering (10%).

### 2.3 Power considerations

Our sample size of 48 raters (12 per gender‐by‐age category) was chosen to achieve the aims of the larger study,^18^ but we anticipated it would provide adequate power to detect meaningful associations between our independent variables and clip persuasiveness. Our dependent variable (perceived persuasiveness) was measured on a 1 to 5 scale; we considered that a 0.5‐point difference would be clinically meaningful. The 1152 clip ratings (48 clips, each rated by 24 raters) were not independent, because of clustering due to rater, clip (story), and storyteller. We used the design effect (DE) to translate this planned number of ratings into an ‘effective sample size’ of independent observations that can be used for standard power calculation.^19^ DE depends on the intraclass correlation coefficient (ICC), calculated as a ratio of the variance of interest to total variance.^20^ Assuming the sum of all variances of interest (i.e., between raters, clips, and storytellers) accounts for 50% to 70% of total variance (i.e., an ICC between 0.5 and 0.7), the effective number of ratings ranges from 92 to 67. Based on data from similar scales and patient populations,^16^ we conservatively estimated the standard deviation (SD) for persuasiveness to range from 0.5 to 0.7. Under these assumptions, our analyses would have at least 87% power to detect a slope of 0.5 points (ie if a 1‐point increase in engagement were associated with an increase of at least 0.5 points in persuasiveness) for a two‐sided significance test and an alpha of 0.05.

When examining the effects of gender and age concordance, our sample was designed so that half of the ratings were from raters who had the same gender and/or age as the storyteller and half were from raters who did not. Using the same strategy as above, for an ICC of 0.7, the effective sample size is 33 per group and the power to detect a 0.5‐point difference in persuasiveness between the clips rated by age‐ or gender‐concordant raters and those rated by age or gender non‐concordant raters is at least 82%. The power would be >92% if the ICC was 0.5.

### 2.4 Statistical analyses

Given the large number of topics and the substantial proportion of clips that included multiple topics, we used latent class analysis to classify clips into homogenous classes, or themes, based on the combination of topics that each clip included. We performed a latent class analysis to simplify how we analysed clip content and reduce the chances of type I error. We examined models with two to five themes (classes) and selected the optimal number of themes based on the proportion of clips assigned to each class, interpretability of results and several goodness‐of‐fit criteria (Bayesian information criterion, Akaike information criterion, entropy, and Lo‐Mendell‐Rubin and parametric bootstrapped likelihood‐ratio tests).^21^, ^22^ The highest posterior probability from the optimal latent class analysis model was used to assign each clip to one theme. For statistical analyses, each theme was operationalized as a binary variable indicating whether a particular theme was present in each clip. Themes were mutually exclusive; each clip was assigned to only 1 theme.

We started by fitting separate linear mixed‐effects models to examine unadjusted (bivariate) associations between each independent variable (patient engagement with the clip, age concordance between rater and storyteller, and gender concordance between rater and storyteller) and our dependent variable (perceived persuasiveness).^23^ We then added all 3 independent variables into a single multivariable model. Three random effects (for storyteller, clips nested within storyteller and rater) were included to account for raters viewing multiple clips and clips clustered within storyteller.

We also examined associations between covariates and our dependent variable. We first analysed each covariate separately in unadjusted linear mixed‐effects models controlling only for raters viewing multiple clips and for clips being nested within storytellers. We then added covariates with *P* < .2 in unadjusted analyses, one at a time, to the multivariable model containing our three independent variables and retained only those that were significantly associated with perceived persuasiveness in the final model. Finally, to evaluate the relative impact of storyteller vs story on perceived persuasiveness, we compared the ICCs for the storyteller vs the video clip to examine the proportion of total variance of the dependent variable explained by each random component of our mixed‐effects model.^20^

We used Mplus version 8^24^ to perform latent class analyses. All other analyses were implemented using SAS version 9.4 (SAS Institute Inc). All tests were two‐sided, with an alpha of 0.05. Due to our relatively small sample size, we did not explore mediators or moderators of perceived persuasiveness or examine statistical interactions.

## 3 RESULTS

As planned, we recruited 48 raters (12 for each gender‐by‐age category); 79% of patients who were screened and eligible for inclusion agreed to participate. Table 1 shows rater characteristics.

We chose the three‐class model as the best‐fitting latent class analysis model for the clip topics in our data. Table 4 shows the item response probabilities for each topic for each theme identified using this model. Based on the clip topics included in each theme, we named the themes *Problems with opioids*, *Daily functioning* and *Opioid access*. As conveyed in Table 4, clips assigned to the *Problems with opioids* theme were most likely to include the topics reasons for tapering, opioid risks and managing opioids; clips assigned to the *Daily functioning* theme were most likely to include the topics getting through the day and managing pain; and clips assigned to the *Opioid access* theme were most likely to include the topics communicating with clinicians and managing opioids. Of the 48 clips, 12 (25%) were assigned to *Problems with opioids*, 27 (56%) to *Daily functioning* and 9 (19%) to *Opioid access*. Assignment probabilities were excellent for all 3 themes (means of 0.99, 0.98 and 0.98, respectively).

**TABLE 4:** Item response probabilities of topics given a latent class for the best‐fitting latent class analysis model^a^

	Clip theme (class)		
	Problems with opioids	Daily functioning	Opioid access
Clip topic			
Reasons for tapering			
Yes	**0.83**	0.25	0.23
No	0.17	0.75	0.77
Opioid‐related risks			
Yes	**1.0**	0.14	0
No	0	0.86	1.0
Fears about tapering			
Yes	0	0.08	0.21
No	1.0	0.92	0.79
Benefits of tapering			
Yes	0	0.19	0
No	1.0	0.81	1.0
Communicating with clinician			
Yes	0	0.08	**0.91**
No	1.0	0.92	0.09
Managing pain			
Yes	0.11	**0.88**	0
No	0.89	0.12	1.0
Managing opioids			
Yes	**0.67**	0.18	**0.57**
No	0.34	0.82	0.43
Getting through the day			
Yes	0.19	**0.84**	0
No	0.81	0.16	1.0
Support			
Yes	0	**0.41**	0.11
No	1.0	0.59	0.89

^a^ Item response probabilities for the presence of a topic over 0.40 are bolded for emphasis.

Table 5 shows our primary results for both unadjusted and adjusted analyses. In unadjusted analyses of our independent variables, higher patient engagement was associated with higher perceived persuasiveness and age concordance was associated with lower perceived persuasiveness. However, gender concordance was not significantly associated with perceived persuasiveness. When all 3 independent variables were examined in the same multivariable model, only patient engagement remained significantly associated with perceived persuasiveness.

**TABLE 5:** Factors associated with perceived persuasiveness

	Unadjusted analysis^a^			Final model^b^		
	Coefficient	95% CI	*P* value	Coefficient	95% CI	*P* value
Independent variable						
Engagement with clip	0.47	0.40 to 0.53	<.001	0.46	0.39 to 0.53	<.001
Age concordance	−0.13	−0.23 to −0.02	.02	−0.08	−0.18 to 0.02	.11
Gender concordance	0.01	−0.10 to 0.12	.84	−0.02	−0.12 to 0.08	.71
**Covariate**						
Rater						
Attitude about opioid tapering^c^	0.42	0.04 to 0.81	.03			
Belief about effectiveness	−0.10	−0.29 to 0.10	.33			
Belief about side‐effects	0.15	−0.10 to 0.41	.24			
Clip						
Clip duration (10s)	0.05	−0.03 to 0.13	.24			
Clip themes^d^						
Problems with opioids	0.27	0.05 to 0.49	.02	0.26	0.03 to 0.49	.03
Daily functioning	−0.22	−0.42 to −0.03	.03			
Opioid access	0.03	−0.24 to 0.29	.83			

^a^ From linear mixed‐effects regression models fitted separately for each independent variable and controlling only for raters viewing multiple clips and for clips nested within storytellers.

^b^ From linear mixed‐effects regression models controlling for all listed independent variables and covariates that have coefficients listed in the second column and accounting for raters viewing multiple clips and for clips being nested within storytellers.

^c^ Analysed as a binary variable: disagree or strongly disagree with desire to taper (reference) vs agree, strongly agree or already tapered.

^d^ Clip themes were analysed as binary variables indicating whether each clip contained the specific theme or not (reference).

Among the covariates examined, beliefs about opioid tapering (but not beliefs about opioid effectiveness, opioid‐related side‐effects or clip duration) were significantly associated with perceived persuasiveness in unadjusted analyses. Patient raters who endorsed a desire to taper or had already tapered rated clips as significantly more persuasive than raters who disagreed with a desire to taper. Two of the 3 themes—*Problems with opioids* and *Daily functioning*—were also associated with perceived persuasiveness (*P* < .2) in unadjusted analyses and so were considered for inclusion in the final model (Table 5).

When added to the multivariable model containing all three independent variables, only one covariate—the clip theme *Problems with opioids*—remained significantly associated with perceived persuasiveness and was retained in the final model. Clips with stories that discussed problems with opioids were perceived as significantly more persuasive (coefficient = 0.26; 95% CI 0.03, 0.49, *P* = .03) than clips assigned to either of the other two themes. Of the 3 independent variables, only patient engagement remained significantly associated with perceived persuasiveness (coefficient = 0.46; 95% CI 0.39, 0.53, *P* < .001) in the final model. The ICC was 6.5% for the clip level and 0% for the storyteller level in the empty model and 7.9% for the clip level and 0% for the storyteller level in the final mixed models.

## 4 DISCUSSION AND CONCLUSION

### 4.1 Discussion

This study examined factors correlated with perceived persuasiveness of video‐recorded narrative clips collected during a larger project on opioid tapering. Patients were involved in this study both as storytellers (in each clip) and as raters assessing perceived persuasiveness. We found that higher rater engagement with brief narrative clips was strongly and significantly associated with greater perceived persuasiveness. In contrast, age concordance and gender concordance between patient raters and storytellers were not significantly associated with clips’ perceived persuasiveness in multivariable models. These findings suggest that highly engaging stories or vignettes are likely persuasive to patients regardless of the storytellers’ age and gender or patients’ age and gender. One possible explanation of this finding is that, while a patient's identification with the storyteller is a key element of persuasion in narrative persuasion theory,^4^ being of the same gender or age group, by itself, is often not sufficient to make patients identify with storytellers in narrative videos. This interpretation is consistent both with an older review that found similarity between participants and storytellers was not typically associated with persuasiveness,^8^ and with the findings from the recent study by Ooms et al.^9^ That study found limited effects of age and gender concordance on persuasion; however, when the authors fit structural equation models, measurements of perceived similarity did contribute significantly to persuasiveness. If confirmed in other studies, an implication of this interpretation is that health researchers should consider multiple factors associated with patient‐storyteller identification (eg storyteller authenticity, storyteller context) rather than just simple demographics when selecting storytellers for narrative videos.

One unexpected finding was that raters who agreed or strongly agreed that tapering was beneficial found clips to be much more persuasive than raters who disagreed that tapering was beneficial. An implication of this finding is that the video produced as part of the larger project may be more persuasive to patients who are at least open to the idea of tapering opioids vs patients who believe tapering will lead to worse pain control. This finding may relate to the consensus from prior reviews that it is particularly difficult to persuade patients to stop harmful or unwanted behaviour, compared with taking on or initiating healthy behaviour.^7^, ^8^ Our finding that clips assigned to the theme *Problems with opioids* were more persuasive than other clips suggests that using stories that recount patients’ rationale for tapering, including concerns about opioid‐related risks, is a promising strategy for future interventions related to opioid tapering and, potentially, other interventions focused on behavioural cessation.

Notably, in this analysis of 48 clips featuring 7 different storytellers, storyteller identity did not independently explain *any* of the variance in perceived persuasiveness (ie ICC for storyteller was 0). Raters and storytellers were recruited from the same patient population using the same procedures, so one potential explanation for this finding is that persuasiveness was driven by raters’ identification with the clinical scenarios described in the clips or problems with opioids described by the storytellers rather than by the demographic characteristics of the storytellers. These findings indicate that narrative content also impacts the perceived persuasiveness of patient narratives.

Our study has some limitations. The small number of unique storytellers in the clips and sample size may have limited our ability to detect small effects or to reliably estimate the ICC for storyteller; however, our sample was balanced to optimize our ability to evaluate the effects of age and gender concordance. We could not examine racial concordance because most raters and storytellers identified as white. Our decision to measure whether raters perceived that clips were likely to be persuasive for others as the dependent variable rather than whether clips were persuasive to raters themselves was consistent with the larger project's goal, but measuring the latter as the dependent variable might have generated different results. The clips used in this study were shorter than the recorded and written narratives typically analysed in the studies of narrative transportation theory. However, patient engagement with stories and attitudes towards storytellers often form quickly, and ratings based on short video clips of the kind used in this study tend to be highly correlated with ratings based on longer recordings.^25^

### 4.2 Conclusions

When viewing brief video‐recorded narrative clips about opioid tapering, greater patient engagement with the clip, but not age or gender concordance with the storyteller, was associated with greater perceived persuasiveness for promoting opioid tapering. Additional studies are needed in this area; however, our findings suggest that patient engagement with a story that is clinically relevant to the patient is likely more important for determining persuasiveness than are demographic similarities between patient and storyteller.

Results from this study are relevant to clinicians, researchers and health educators planning education videos or interventions to promote health behaviour change. Videos and interventions that incorporate patient narratives that are clinically relevant and highly engaging to patients are likely to be persuasive regardless of storyteller demographics. Conversely, using different storytellers to target specific patient demographic groups may not substantially increase overall persuasiveness.

## CONFLICTS OF INTEREST

No authors have any conflicts of interest to disclose.

## AUTHOR CONTRIBUTIONS

Henry conceived and designed the study, obtained funding, planned and interpreted the analysis results, wrote the first draft of the manuscript and approved the final submission. Feng planned the analysis, edited the manuscript for critical content and approved the final submission. Verba collected the data, edited the manuscript for critical content and approved the final submission. Kravitz interpreted the analysis results, edited the manuscript for critical content and approved the final submission. Iosif planned and conducted the analysis, interpreted the analysis results, edited the manuscript for critical content and approved the final submission.

## ETHICAL APPROVAL

This study was approved by the University of California Davis Institutional Review Board (protocol number 897480).

## Supporting information

Video S1

Video S2

## Data Availability

A de‐identified version of the statistical data set used in this study is available from the corresponding author upon reasonable request. Video clips used in this study are not available for additional research because they cannot be de‐identified.
